# Are new ideas harder to find? A note on incremental research and *Journal of Cheminformatics*’ Scientific Contribution Statement

**DOI:** 10.1186/s13321-023-00798-6

**Published:** 2024-01-15

**Authors:** Barbara Zdrazil, Rajarshi Guha, Karina Martinez-Mayorga, Nina Jeliazkova

**Affiliations:** 1 European Molecular Biology Laboratory, European Bioinformatics Institute (EMBL-EBI), Wellcome Genome Campus, Hinxton, Cambridgeshire CB10 1SD UK; 2 Vertex Pharmaceuticals, 50 Northern Ave, 02210 Boston, MA USA; 3 Institute of Chemistry, National Autonomous University of Mexico, Campus Merida, Merida-Tetiz Highway, Km. 4.5, Ucu, Yucatan, Mexico; 4 Ideaconsult Ltd, 1000 Sofia, Bulgaria

 In the field of cheminformatics, technological advancements in recent times include, e.g., the way chemical information is being represented for large scale screening and *de novo* drug design. Especially, chemical language models originating from natural language processing offer new opportunities for molecular design [1].

However, for science in general and compared to past decades, recent paucity of transformative ideas has been noticed [2]. While there are many explanations for observed technological stagnation, in pharmaceutical R&D, a productivity crisis was already noted ~20 years ago [3, 4]. An often stated scientific/technological reason for stagnation in pharmaceutical R&D, is the “low hanging fruit” problem. That is, the easier-to-tackle problems have been solved already and that what remains are the more complex and more challenging problems (diseases) [5]. Other possible explanations for declining research productivity might be a shift to more “defensive R&D”, which could be a direct consequence of R&D resources being diverted away from risk-taking by investors, managers, and entrepreneurs to instead fulfil regulatory requirements. Instead of fueling innovation, monetary resources are used to keep “old” products on the market [5].

At the same time, we do observe a trend that more and more papers are being published in scientific journals or on preprint servers [6]. In line with this observation, also more data, methods, and models are being made available in the public domain (through publications and/or platforms such as Zenodo [7], GitHub [8], and Hugging Face [9]). As an effect, researchers are often facing a situation of information-overload with the luxurious problem of filtering out the real innovative contributions, that aren’t just incremental improvements of existing ones.

From a publisher’s perspective, every research paper should be regarded as an attempt to contribute new ideas and/or refine old ones. In Cheminformatics, we have observed a few phases of new methodological developments/inventions with consequent iterations of incremental improvements. Examples include (but are not limited to) molecular representation [10], descriptors for QSPR modeling/ML [11, 12], molecular docking algorithms [13], or more recently the development and refinement of generative AI algorithms [14, 15].

The Editors of the Journal of Cheminformatics do not judge articles based purely on scientific novelty. Rather, we consider aspects such as utility and availability, and the contribution itself, along with notions of novelty.

The Scientific Contribution Statement is our attempt to give space to a declaration by authors regarding the contributions made in their research. The authors should use a maximum of three sentences to specifically highlight the scientific contributions that advance the field and what differentiates their contribution from prior work on this topic (https://jcheminf.biomedcentral.com/submission-guidelines/preparing-your-manuscript/research). It should be regarded by authors as an opportunity to highlight their scientific contribution(s) rather than as a burden or additional request by the Editors of J. Cheminform. Such declaration(s) about contributions and novelty have always been part of the scientific publication process—albeit in a more convoluted or scattered way, as there usually isn’t a specific section in a paper dedicated to such declarations. We therefore started to make this vital information more accessible by assigning it a fixed section (namely the Abstract of a paper).

We hope that this amendment will not only help us as Editors when assessing a paper submitted for consideration, but equally the members of our scientific community— reviewers and readers.

## References

[1] Grisoni F (2023). Chemical language models for de novo drug design: challenges and opportunities. Curr Opin Struct Biol.

[2] Bhaskar M (2021) Human frontiers: the future of big ideas in an age of small thinking

[3] Pammolli F, Magazzini L, Riccaboni M (2011). The productivity crisis in pharmaceutical R&D. Nat Rev Drug Discov.

[4] Laermann-Nguyen U, Backfisch M (2021). Innovation crisis in the pharmaceutical industry? A survey. SN Bus Econ.

[5] Cockburn IM (2006). Is the pharmaceutical industry in a productivity crisis?. Innov Policy Econ.

[6] Rawat S, Meena S (2014). Publish or perish: where are we heading?. J Res Med Sci.

[7] https://zenodo.org/

[8] https://github.com/

[9] https://huggingface.co/

[10] Wigh DS, Goodman JM, Lapkin AA (2022). A review of molecular representation in the age of machine learning. WIREs Comput Mol Sci.

[11] Balaban AT, Meyers RA (2009). Drug Design, Molecular descriptors in. Encyclopedia of complexity and Systems Science.

[12] Sahoo S, Adhikari C, Kuanar M, Mishra BK (2016). A short review of the generation of Molecular descriptors and their applications in quantitative structure Property/Activity relationships. Curr Comput Aided Drug Des.

[13] Torres PHM, Sodero ACR, Jofily P, Silva-Jr FP (2019). Key topics in molecular docking for drug design. Int J Mol Sci.

[14] Bandi A, Adapa PVSR, Kuchi YEVPK (2023). The Power of Generative AI: a review of requirements, models, input–output formats, evaluation Metrics, and challenges. Future Internet.

[15] Shokrollahi Y, Yarmohammadtoosky S, Nikahd MM et al (2023) A comprehensive review of generative AI in healthcare

